# Supplementary material for: Age of initiation of cigarillos, filtered cigars and/or traditional cigars among youth: Findings from the Population Assessment of Tobacco and Health (PATH) study, 2013–2017
Source: PLoS One. 2020 Dec 9;15(12):e0243372. doi: 10.1371/journal.pone.0243372 (PMC7725294; doi:10.1371/journal.pone.0243372)
Supplement: Attachment — Submitted filename: Response 10_12_2020.docx [file pone.0243372.s001.docx]

AGE OF INITIATION OF CIGARILLOS, FILTERED CIGARS AND/OR TRADITIONAL CIGARS AMONG YOUTH: FINDINGS FROM THE POPULATION ASSESSMENT OF TOBACCO AND HEALTH STUDY, 2013-2017

We want to thank the reviewers for the excellent comments which help us to make this paper stronger. Our answers are below after each comment.

**Data Availability Statement**: All the data from waves 1-4 are available from the Population Assessment of Tobacco and Health (PATH) Study [United States] Restricted-Use Files. Inter-university Consortium for Political and Social Research [distributor], 2020-06-24. <https://doi.org/10.3886/ICPSR36231.v25>.

Data are available from <https://www.icpsr.umich.edu/web/NAHDAP/studies/36231>

This is in reference #16 in the current version of the manuscript. The website is shown as a footnote in all tables to highly the location of the data.

**Reviewer #1:** 

*Page 7, line 118. Because of demographic differences in use of cigars as blunts (Cullen, J., Mowery, P., Delnevo, C., Allen, J. A., Sokol, N., Byron, M. J. and Thornton-Bullock, A. Seven-year patterns in US cigar use epidemiology among young adults aged 18-25 years: a focus on race/ethnicity and brand. Am J Public Health. 2011 Oct;101(10):1955-62. doi: 10.2105/AJPH.2011.300209. Epub 2011 Aug 18.), it is important to be clear how these subjects were treated. How specifically did the authors exclude blunt use? The PATH question is “**Have you ever smoked part or all of a cigar, cigarillo or filtered cigar with marijuana in it?” Did the authors exclude all blunt users even if they had also been cigar users or did they approach this in another way? Please clarify this question for susceptibility, ever use, past 30-day use, and fairly regular use.*

**Response:** The reviewer is correct that PATH has this question: “Have you ever smoked part or all of a cigar, cigarillo or filtered cigar with marijuana in it?”. However, this is not the variable we used for our analyses. We have clarified this in our new version of the manuscript. PATH asked to participants “Have you ever smoked a traditional cigar, cigarillo, or filtered cigar, even one or two puffs?” then three derived variables are included in the restricted datasets describing ever cigarillo, ever filtered cigar and ever traditional cigar users (i.e. R01R_Y_EVR_GRILLO, R01R_Y_EVR_GFILTR, and R01R_Y_EVR_GTRAD) at each wave “excluding blunt-only users”. We clarified to the readers that for the estimation of the age of initiation of the three outcomes: any cigar product, cigarillos and filtered cigar, and traditional cigars, the analyses included blunt and non-blunt users and excludes blunt-only users. In addition, the FDA does not have regulatory authority over marijuana use, and we have been advised by tobacco researchers in the past to refrain from examining marijuana use. Please refer to lines 136-139, 173-176 in methods and lines 554-566 in the discussion.

*Page 7, line 120. The authors state that other studies combined cigarillo and filtered cigar use. But, why, for this specific study, was it necessary and advantageous to combine cigarillo and filtered cigar use? The demographics of use of these two classes of cigars are quite different. Cigarillos are widely used as blunts and filtered cigars are used as substitutes for cigarettes. It is also possible that age of initiation may be different. Were there data that suggested that these data from cigarillo and filtered cigars were equivalent and could be readily combined in the context of this study’s objectives?*

**Response:** We combined cigarillos or filtered cigars into one category because in youth, the proportions for cigarillo and filtered cigar were small (especially for past 30-day and fairly-regular use) and to increase the power of this study, we combined the two into one group, as used in the other studies. Other PATH publications end up reporting blank spaces as it was not possible to obtain individual estimates (i.e. Villani et al. (2017) Flavored Tobacco Product Use in Youth and Adults: Findings From the First Wave of the PATH Study (2013–2014) Am J Prev Med. 2017 August ; 53(2): 139–151). We added these explanations in page 7 lines 148-143 in the Measures subsection of the manuscript. In addition, we described the similar characteristics of cigarillos and filtered cigars including brands and the differences with the traditional cigars in lines 63-79.

*Page 8, line 140 and following. The discussion of methods for ever use, past 30-day use, and fairly regular use are very repetitious. These can be combined by listing the three PATH questions and then explaining how the participants were categorized based on their responses without repeating these steps three times.*

**Response:** We agree with you that these steps were repetition. We revised this based on your suggestions and reduced the wording. See lines 163-166, 178-188, 195-203 in document with track changes.

*Page 9, line 173. How did the authors address dual use? Were dual users included or excluded? Were these exclusive cigar users or were cigar/cigarette dual users also included in the test population? If so, how many of each category were excluded due to dual users?*

**Response:** In the original lines 173-179, we did not mention cigarette use in the inclusion/exclusion criteria for our analysis. We started with never users of cigar products but it is possible that never users of cigar products may have use cigarettes or other tobacco products at the first wave of participation into PATH. Table 1 shows the number of tobacco products other than cigars. Future analyses of the age of initiation of cigar products may explore the influence of cigarette use before cigar product initiation. This limitation was added in lines 562-566.

*Page 12, line 230. Table 2 and Figures 1-3 are sowing the same data. Either the Tables or the Figures should be in Supplemental instead.*

**Response:** We agree with you that there are some overlaps between Table 2 and Figures 1-3. However, the tables are easier to read by reporting numbers of the hazards and confidence intervals for some specific ages, while the figures provide more information (e.g., the hazards at different ages that are not listed in the tables). So, we choose to report both the tables and Figures to provide more information for the reader.

*Page 20, line 333. But there may have been differences by race/ethnicity if the authors had analyzed cigarillo and filtered cigars separately.*

**Response:** Potentially they may have been differences by race/ethnicity if we had analyzed cigarillo and filtered cigars separately. However, the sample size for each one alone was not sufficient to obtain convergence in the estimates, for this reason we had to combine cigarillo or filtered cigars as an outcome. Please see our earlier response regarding this issue.

*Page 25, line 448. Since age of initiation of traditional cigar use is much higher than for cigarillo and filtered cigars (manuscript lines 389-390), what does that suggest for FDA regulation of these products? Should they be regulated differently? If FDA were to use a* *youth-protection rationale for regulating cigars, should they include traditional cigars in the regulation?*

**Response:** Thank you for pointing this out. We have added this recommendation in the discussion in lines 494-495. Prevention campaigns for youth should start for cigarillos and filtered cigars at earlier ages (i.e. before 15-16 years old) while campaigns for traditional cigars can be tailored before 16-17 years of age.

*Page 26, line 460. Here the authors describe the reason they should be clear about their rationale and methods for excluding blunt users in this study.*

**Response:** Please refer to lines 136-139, 173-176 in methods and lines 554-566 in the discussion.

*Page 27, line 473. The authors need to discuss possible differences in the meaning of “fairly regular” between filtered cigar and traditional cigar use. Because traditional cigars are often used rarely (once per week or once per month), the understanding of “fairly regular” may be different from “fairly regular” use of filtered cigars or cigarillos.*

**Response**: Similar to traditional cigars, cigarillo and filtered cigar products are also used occasionally as noted in the discussion. The PATH study did not define “fairly regular”, participants choose to report “fairly regular” if they think they were “fairly regular users”. Combining the variable of “fairly regular” use with the number of times that was used will required to combine the number of times that the participants would consider the use of the cigars. PATH measured the following questions:

a) “How many [each cigar product is replaced here] have you smoked in your entire life?” with responses: 1 or more puffs but never a whole one, 1, 2 to 10, 11 to 20, 21 to 50, 51 to 99, 100 or more;
b) “When was the last time you smoked a [each cigar product is replaced here], even one or two puffs?” with responses: Earlier today, Not today but sometime in the past 7 days, Not in the past 7 days but sometime in the past 30 days, Not in the past 30 days but sometime in the past 6 months, Not in the past 6 months but sometime in the past year, 1 to 4 years ago, 5 or more years ago;
c) “In the past 30 days, on the days you smoked a [each cigar product is replaced here], how many [each cigar product is replaced here]s did you smoke per day? with responses: Less than 1 per day, 1 per day, 2 to 5 per day, 6 to 10 per day, 11 to 20 per day, 6 More than 20 per day.

Regrettable that frequent variable is not available in PATH for those participants who reported “fairly regular” use. Additional research is needed to understand how users of all cigar products interpret “fairly regular” use and this was also beyond the scope of this manuscript.

*Minor correction:

Page 6, line 111 – Correct to keep commas consistent.*

**Response:** We have revised this accordingly.

Reviewer #2: Understanding patterns of cigar use initiation by age, race, gender, and product type is critical to develop appropriate prevention efforts and tobacco control policies. While this is an important study that contributes valuable knowledge to the field, there are several areas that can be improved. Namely, the importance of differentiating cigar product type can be better described in the Introduction and Discussion – the results indicated that different epidemiological patterns emerged when making this distinction, so this argument should be highlighted more prominently. Additionally, some methodological approaches can be more clearly described. Below are specific recommendations to improve the manuscript.

INTRODUCTION
*- The authors acknowledged an important limitation of prior studies on cigar use, which often collapse different types of cigar products into one broad category. However, the importance of studying cigar use by type was not well-established in the Introduction. Why is it so critical to investigate these differences (e.g., are there differences in health risks, addiction potential, appeal, price, etc.)? This would make a more compelling case for the study.*

**Response:** We appreciate the insight, we have added the a more compelling case for the study differentiating the appealing and cost reasons and others for cigarillo/filtered product use among youth in lines 63-79. Also in lines 112-114, we emphasized the need to reveal differences in ages of initiation by demographic characteristics for the purpose of tailoring prevention campaigns.

*- Similarly, it would be helpful to briefly describe how these 3 products differ in addition to size. For example, large cigars are generally used less frequently and are not commonly inhaled, some cigarillos are primarily used as blunts to smoke marijuana, filtered cigars are often used as cheaper cigarette substitutes. This is mentioned in the Discussion, but this context seems important to describe upfront.*

**Response:** Thank for you the suggestion. This has been added to the introduction. Please see lines 63-79.

METHODS
*- The authors created a combined category for cigarillo and filtered cigar use, noting that this is “consistent with other published studies.” The collapsing of these categories is at odds with the authors’ criticism of prior studies detailed in the introduction. Moreover, the ways in which these products are regulated and used differ: little filtered cigars, which are sometimes indistinguishable from cigarettes, are usually sold in packs of 20 and are taxed at the same rate as cigarettes at the federal level. Cigarillos, however, vary more widely in their flavor offerings, pack sizes, cost, and consumer profiles. If the data are available, it seems like a missed opportunity to not distinguish between these products.*

**Response:** We agree with the reviewer that we would have love to report cigarillos an filtered cigars individually after 4 waves of follow up but the sample size for each one alone was not sufficient to obtain convergence in the estimates, for this reason we had to combine cigarillo or filtered cigars as an outcome. Please see our earlier response regarding this issue. We expect that when future waves of PATH are released we will be able to report the estimates for cigarillo alone and traditional cigar alone.

*- The last sentence in the “Ever use” section repeats what was said in the second sentence and can be removed. This is true for the “Past 30 day use” and “Fairly regular use” sections, as well.*

**Response:** We have removed the duplicated information and reduced substantially the description but given the questions related to blunt we had to still explain to the readers the derived versus the questions asked to the PATH participants. See updated version in lines 163-167.

*- The description about how age of initiation was determined (i.e., adding the number of weeks between waves to the participant’s age) was unclear. Without knowing their birthdate, how is this used to determine age? For example, two participants who are 16-years old at wave 1 could be almost a year apart in age. A combination of birthdate plus dates of survey waves would be more precise. Is this information available? More clarification is needed in this section.*

**Response:** Unfortunately, the restricted PATH data does not provide the date of birth of participants for researchers use. It only provided the age in years. For this reason, the age in years and the number of weeks between surveys is the closest estimate for the exact age of initiation of the cigar products. We clarified it in page 10 lines 226-227.

*- Another limitation is that it is not possible to determine the age when participants actually initiated each category of use (at least in my understanding of how these measures were described). For example, if in the second wave a participant states that they are an ever user, past 30 day user, or used fairly regularly, they may have “initiated” those behaviors immediately after wave 1, at the mid-point between waves, or just before wave 2. Since they are only asked about these behaviors during their interviews – but are not asked when these behaviors actually first occurred - how can the authors determine the age when these behaviors first took place?*

**Response:** We agree with you that we cannot determine the exact age when participants actually initiate each cigar product. That is why we had to implement interval-censored survival methods. In the interval-censored survival methods, we only need to know an interval in which the event (each cigar initiation) occurs, and it is not necessary to know the exact initiation time. For example, if a participant reported ever use at wave 2 (t2), but not at wave 1 (t1), then we know that the initiation occurs in the interval (t1, t2], but we do not know the exact time. The interval-censored survival methods just handles this kind of data. References are provided to the readers on how the methodology provides unbiased estimates.

RESULTS
*- Because of the sample size issues for the “fairly regular” use variable (i.e., unstable estimates for traditional cigar use across all ages and across most ages for the other cigar variables), it might be worth considering dropping this variable from the analyses. This might also help pare down the paper, which has a fairly wide scope.*

**Response:** Thank you for the suggestion. Although there is sample size issue in some ages, the fairly regular use results might be of interest for some readers and we already have invested many hours in obtaining the estimates for this reason we would keep it in the results.

TABLES
*- The title for Table 1 seems disjointed and is a bit confusing to interpret.*

**Response:** We revised the title of Table 1 to make it more readable.

*- In Table 1, it might be more interpretable to present the percentage of participants who use another tobacco product, instead of the average number of products used (which are all <1).*

**Response:** We removed the mean estimates and replaced after new disclosure the percentage of participants who use other tobacco products grouped in 0, 1, and 2 other tobacco products.

*- In Table 5, some of the numbers for “fairly regular use” seem too high. For example, the cumulative incidence by 18 years old is over 50% for most groups, which is substantially higher than ever or past 30 day use.*

**Response:** We agree with you that the jump maybe due to different interpretations of “fairly regular use when the participants are 17 years old or more by race/ethnicity. When checking table 2 we added the estimates for cigarillos and filtered cigars for “fairly regular” that we omitted by mistake in the able but that it is noticeable in the figures.

*- Moreover, in Table 5, why are only certain categories presented? (e.g., susceptibility only for traditional cigars, fairly regular use only for filtered cigars/cigarillos)*

**Response:** In Table 3, we reported the statistical difference among race/ethnicity for different cigar categories. Only those categories that are significant were reported in Table 5. For those categories that are not significant, we can refer to Table 1 to see the overall age of initiation. This is explained to the readers in lines 258-261.

DISCUSSION
*- There is a typo on page 25, line 444. It should read “2013-2014.”*

**Response:** Thanks for the careful comment. We revised it.

*- The authors should clarify their recommendation to differentiate cigar interventions by behavior (e.g., ever use, past 30 day, “fairly regular”). Traditionally, interventions target primary prevention or cessation. What is meant by this?*

**Response:** We agree with the reviewer and have removed the sentence from the discussion.

*- The authors focus mainly on the filtered cigar/cigarillo findings, and briefly describe findings for traditional cigars, but the observed differences between these two products – especially by race – seem noteworthy and should be discussed in greater detail. White youth are more likely to initiate traditional cigars, whereas Black youth have considerably higher rates of initiation (and at earlier ages) for filtered cigars/cigarillos. The typical use behaviors (e.g., frequency, purpose) and product characteristics (e.g., price, flavorings, packaging size, accessibility, etc.) between these two categories differ, and may influence racial/ethnic disparities in youth tobacco use. Discussing this issue might be one way to justify the importance of analyzing cigar use epidemiology by product type, which the authors set out to do in the Introduction.*

**Response:** We are grateful for this comment, we have incorporated these suggestions in our discussion. Please see page 27, lines 522-527.
