# Supplementary material for: Age of initiation of cigarillos, filtered cigars and/or traditional cigars among youth: Findings from the Population Assessment of Tobacco and Health (PATH) study, 2013–2017
Source: PLoS One. 2020 Dec 9;15(12):e0243372. doi: 10.1371/journal.pone.0243372 (PMC7725294; doi:10.1371/journal.pone.0243372)
Supplement: Attachment — Submitted filename: Response-R2 11_16_2020.docx [file pone.0243372.s002.docx]

AGE OF INITIATION OF CIGARILLOS, FILTERED CIGARS AND/OR TRADITIONAL CIGARS AMONG YOUTH: FINDINGS FROM THE POPULATION ASSESSMENT OF TOBACCO AND HEALTH STUDY, 2013-2017

We want to thank the reviewers for the excellent comments which help us to make this paper stronger. Our answers are below after each comment.

*Have the authors made all data underlying the findings in their manuscript fully available?

The* [*PLOS Data policy*](https://urldefense.proofpoint.com/v2/url?u=http-3A__www.plosone.org_static_policies.action-23sharing&d=DwMGaQ&c=bKRySV-ouEg_AT-w2QWsTdd9X__KYh9Eq2fdmQDVZgw&r=Kb-0IID2d6wtjfUeR0Wf8bHZzWLTzSMekCsYlOSU0uM&m=uyUErlt3V76UMqRZ4iYjmzB2p1G9vlhT2kdv9--lUdI&s=toTaSsGyZpsokRjfYR29ySNO8nuD-lCHcopIWJtFX-o&e=) *requires authors to make all data underlying the findings described in their manuscript fully available without restriction, with rare exception (please refer to the Data Availability Statement in the manuscript PDF file). The data should be provided as part of the manuscript or its supporting information, or deposited to a public repository. For example, in addition to summary statistics, the data points behind means, medians and variance measures should be available. If there are restrictions on publicly sharing data—e.g. participant privacy or use of data from a third party—those must be specified.*

*Reviewer #2: No*

**Response:** We clarified that the PATH data we used for all analyses are restricted data from a third party. Here we include the data availability statement we made for the paper. Interested researchers can apply for the restricted data from the third party directly.

**Data Availability Statement**: All the data from waves 1-4 are available from the Population Assessment of Tobacco and Health (PATH) Study [United States] Restricted-Use Files. Inter-university Consortium for Political and Social Research [distributor], 2020-06-24. <https://doi.org/10.3886/ICPSR36231.v25>.

Data are available from <https://www.icpsr.umich.edu/web/NAHDAP/studies/36231>

This is in reference #18 in the current version of the manuscript. The website is shown as a footnote in all tables to highly the location of the data. We believe with this we comply with the journal policy.

**Reviewer #2:** 

*The authors did a commendable job revising this manuscript, which is considerably clearer and stronger. While the Introduction and Discussion sections are substantially improved, I still think a sentence or two could be added about why it is so important to differentiate cigars by subtype from a health perspective. The authors did a great job describing how the products look different and are used differently, but why is this important for individual/population health? One thought is that smaller cigars are generally used more frequently and are often inhaled, while traditional cigars are usually not inhaled and are used more infrequently. This could potentially influence their addictive potential or even their health risks. Two minor comments are listed below:*

**Response:** We thank the reviewer for the excellent comments. Based on your comments, we added the following sentence in lines 79-82 for further clarification:

“From the health perspective, cigarillos or filtered cigars are generally used more frequently in youth and are often inhaled, which may increase young smokers’ risk for addiction to nicotine and/or poor health outcomes[10, 11]. However, the traditional cigars are usually not inhaled and are used less frequently in youth.”

*Pg. 4, lines 67-71: The second part of this sentence (i.e., “socializing when smoking traditional cigars was reported in 2013-2014 with the highest proportion for traditional cigar use exhibited among adults”) was unclear. The sentence would benefit from restructuring.*

**Response:** We have restructured this sentence to make it clear.

*Pg. 7, lines 140-141: The sentence “PATH excluded ‘blunt-only’ users” makes it sound like these individuals were excluded from the PATH study. Please clarify that PATH created derived variables for cigar product use, and that blunt-only users were excluded from these variables.*

**Response:** Yes, we agree with the reviewer and we clarified this sentence based on this suggestion. Thank you
